# *Agrobacterium*-Mediated Transformation of the Recalcitrant *Vanda* Kasem's Delight Orchid with Higher Efficiency

**DOI:** 10.1155/2014/583934

**Published:** 2014-04-08

**Authors:** Pavallekoodi Gnasekaran, Jessica Jeyanthi James Antony, Jasim Uddain, Sreeramanan Subramaniam

**Affiliations:** School of Biological Sciences, Universiti Sains Malaysia (USM), 11800 Minden Heights, Penang, Malaysia

## Abstract

The presented study established *Agrobacterium*-mediated genetic transformation using protocorm-like bodies (PLBs) for the production of transgenic *Vanda* Kasem's Delight Tom Boykin (VKD) orchid. Several parameters such as PLB size, immersion period, level of wounding, *Agrobacterium* density, cocultivation period, and concentration of acetosyringone were tested and quantified using *gusA* gene expression to optimize the efficiency of *Agrobacterium*-mediated genetic transformation of VKD's PLBs. Based on the results, 3-4 mm PLBs wounded by scalpel and immersed for 30 minutes in *Agrobacterium* suspension of 0.8 unit at *A*_600nm_ produced the highest GUS expression. Furthermore, cocultivating infected PLBs for 4 days in the dark on Vacin and Went cocultivation medium containing 200 **𝜇**M acetosyringone enhanced the GUS expression. PCR analysis of the putative transformants selected in the presence of 250 mg/L cefotaxime and 30 mg/L geneticin proved the presence of *wheatwin1*, *wheatwin2*, and *nptII* genes.

## 1. Introduction

Among the horticultural and floral crops, orchids are outstanding in many ways, like diverse shapes, forms, and colours. Orchids are marketed both as potted plants and as cut flowers and their production has increased in recent years [1–3]. Among the orchids, the genus* Vanda *is known to produce large, colourful, and stunning orchids with blooming frequencies of six or more times per year and lasting inflorescences that remain on the plant for between four and eight weeks [4].* Vanda *Kasem's Delight orchid (VKD) has commercial value and priced for the hybrid's diverse shapes, forms, and colours [5]. The aesthetic value of VKD contributes to its commercial value as a cut flower and potted plant. Thus, it is important to produce VKD with economically important traits such as disease and pest resistances, novel flower colours, and tolerances to environmental stresses such as low temperatures and low light intensities. However, it is difficult to produce such varieties through conventional breeding techniques which are based on sexual crossing due to the long generation time and lack of useful genetic variability [6]. Thus, extensive effort is now being made to genetically modify the economically important traits of VKD. Furthermore, establishment of transformation methods for VKD is important to understand the role of a specific gene or DNA (probably via gene knockout method) and to manipulate them in* Vanda *orchids [7].

The molecular transformation technique is an alternative approach to introduce specific characteristics into orchid plants, especially for modification of ornamental characteristics such as flowering time, shelf life, flower colour, and architecture [8].* Agrobacterium tumefaciens*-mediated plant transformation has become the most used method for the introduction of foreign genes into plant cells. Orchids have been genetically modified using* Agrobacterium*-mediated transformation including* Dendrobium *[9–11],* Cymbidium *[12, 13],* Phalaenopsis *[6, 14–16], and* Oncidium *[17].* Agrobacterium*-meditated transformation generates high proportion of transgenic plants while the protocol is relatively simple and straightforward with minimal equipment costs [18].

Early detection of plant transformation events is necessary for the optimization of transient and stable gene transfer into a plant genome [19]. For example, the use of the *β*-glucuronidase (GUS) encoding reporter gene (*uidA*) allows histochemical localisation of gene expression [20]. The expression of* uidA* is easily visualized through the activity of de novo synthesized *β*-glucuronidase (GUS), which produces blue colouration of transformed cells by catalyzing the exogenously applied substrate, X-gluc (5-bromo-4-chloro-3-indolyl glucuronide) [20].

During plant genetic transformation, only few cells will receive the foreign gene among the thousands of cells of explants [21]. One of the key factors in production of transgenic plants involves the selection and regeneration of transformed explants containing stably integrated foreign gene. Thus, a selectable marker gene code for a selective agent is introduced simultaneously with the novel foreign DNA [22]. Currently, selection markers such as* nptII*,* hpt,* and* bar* genes (encoding neomycin phosphotransferase, hygromycin phosphotransferase, and phosphinothricin acetyltransferase resp.) are widely used for selection purpose [19, 23, 24].

PR 4 has been reported to be effective in inhibition of the pathogen hyphal growth and reduction of spore germination [25]. Caporale and team isolated and sequenced four PR-4 proteins from wheat kernels, named* wheatwin1* to* wheatwin4*, that inhibit phytopathogenic fungi with a wide host range (*Botrytis cinerea*) and host-specific pathogens (*Fusarium culmorum, F. graminearum*). Since* wheatwin1* has similar amino acid sequence with that of* wheatwin2*, it is speculated that* wheatwin1* might similarly accumulate extracellularly [26]. This allows the PR 4 proteins to hydrolyze chitin which is the major component of fungal cell wall.

In the present study, the influences of single PLB size, degree of wounding, immersion and cocultivation period, bacterial density, and concentration of acetosyringone (AS) in the modified Vacin and Went [27] cocultivation medium were examined. The* Agrobacterium*-mediated transformation procedure designed should facilitate high-throughput transformation of VKD's PLBs for efforts such as T-DNA gene tagging, positional cloning, or attempts at targeted gene replacement.

## 2. Materials and Methods

### 2.1. Plant Materials

**Figure 1:**
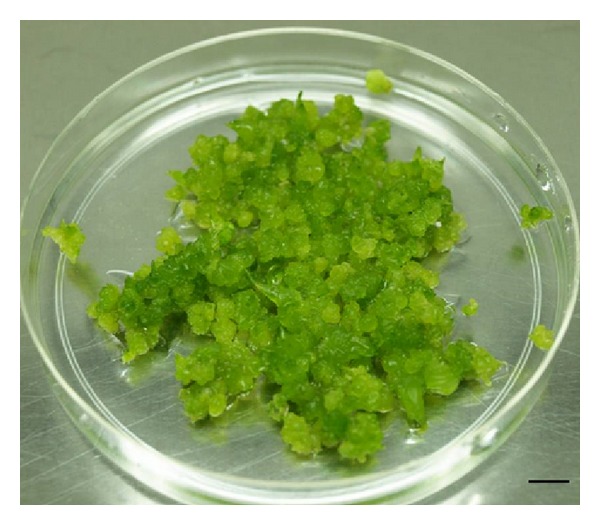
*In vitro* culture of VKD's PLBs. Bar represent 1 cm.

Healthy 12-week-old protocorm-like bodies (PLBs) of* Vanda* Kasem's Delight Tom Boykin (Figure 1) were used as explants for* Agrobacterium-*mediated genetic transformation.

### 2.2. *Agrobacterium tumefaciens* Strains and Plasmid DNA

**Figure 2:**
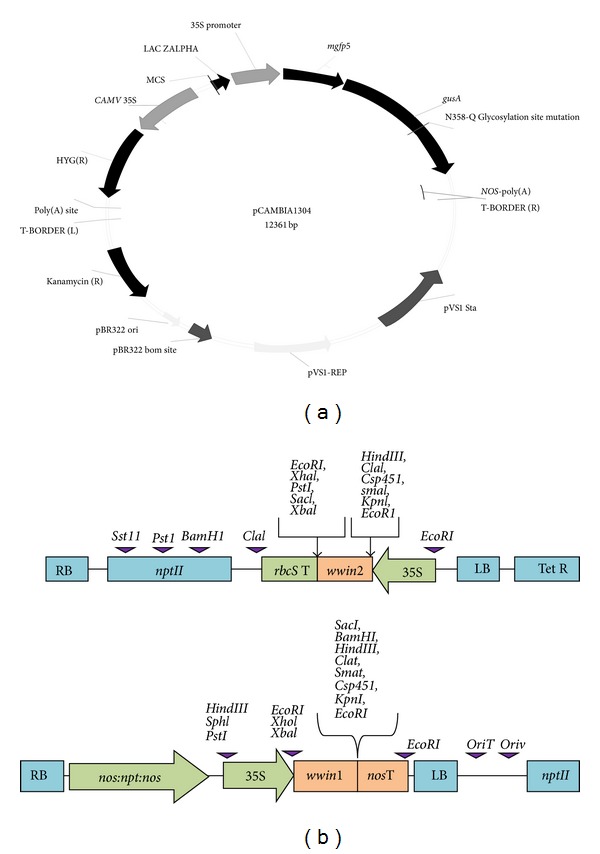
Schematic diagram of the plasmid used for optimization and transformation studies. (a) The binary vector pCAMBIA 1304 (CSIRO, Australia) harboring the reporter* gusA *and* mgfp5 *genes driven by the* CaMV *35S promoter. (b) Recombinant plasmids pW2KY and pW1B1 containing* wwin2* and* wwin1* genes, respectively. RB: right border, LB: left border,* nos *promoter: nopaline synthase promoter,* nos*T: nopaline synthase terminator,* nptII*: neomycin phosphotransferase resistance gene,* nos* poly(A): nopaline synthase polyadenylation signal,* wwin1*: coding region of the PR4,* wwin2*: coding region of the PR4, 35S promoter: cauliflower mosaic virus (CaMV) 35S promoter, and* rbcS *poly(A): ribulose-1,5-bisphosphate carboxylase small subunit gene polyadenylation signal. Relevant restriction sites are also indicated.

*A. tumefaciens *strain LBA4404 harbouring disarmed plasmid pCAMBIA 1304 plasmid with* gusA* and* nptII* genes (Figure 2(a)) was used for optimization of selected parameters involved in* Agrobacterium*-mediated transformation. Plasmid pCAMBIA 1304 was provided byDr. Richard Bretell from CSIRO, Australia. The plasmid driven by* CaMV 35S *promoter contains an intron-interrupted *β*-glucuronidase (*gusA*) gene and the neomycin phosphotransferase II (*nptII*) gene conferring resistance to the aminoglycoside antibiotics such as kanamycin, geneticin, and neomycin. The portable intron in* gusA* gene allows expression of GUS only in transformed plant cells [28].

*Agrobacterium *strains (*A. tumefaciens *strain LBA4404 harbouring plasmid pW1B1 carrying PR4 gene* wwin1*,* nptII* gene;* A. tumefaciens *strain LBA4404 harbouring plasmid pW2KY carrying PR4 gene* wwin2 *and* nptII* gene) (Figure 2(b)) were used for transformation of VKD PLB with optimized condition.* A. tumefaciens *strain LBA4404harboring plasmids pW1B1 and pW2KY was kindly given by Marrina Tucci from National Research Council, Institute of Plant Genetics, Portici, Italy. Bacteria cultures were maintained at −80°C for long-term storage in 70% (v/v) glycerol.

### 2.3. Preparation of Aminoglycoside Antibiotics

Kanamycin, geneticin (G-418), and neomycin were purchased from Sigma Chemical Company. Green and healthy 12-week old PLBs of VKD were subjected to various concentrations of kanamycin, geneticin (G-418), and neomycin. The selection agents were added to the concentrations of 0, 5, 10, 15, 20, 25, 30, 35, 40, 45, and 50 ppm to the modified Vacin and Went media supplemented coconut water and 30% tomato homogenate. The plates were incubated under 16-hour light/8-hour dark photoperiod at 25 ± 2°C. Observations on change of colour and growth and regeneration of explants were done once a week for four weeks. Survived explants were determined based on the colour of explants that remained green.

### 2.4. Optimization of *Agrobacterium* Mediated VKD PLBs Transformation

*A. tumefaciens *strain LBA4404 carrying pCAMBIA1304 was grown on a shaker at 120 rpm and at a temperature of 28°C for 16 hours to an optical density of 0.8 (OD_600 nm  _ = 0.8) in Luria Bertani (LB) medium containing 50 ppm kanamycin. Once the preferred OD is achieved, 100 *μ*M acetosyringone was added to the bacterial suspension culture to increase the virulence. Four- to twelve-week-old healthy green PLBs (PLBs used for explants size optimization) were transferred into* Agrobacterium *suspension for transformation. The PLBs were immersed in* Agrobacterium *cell suspension for 30 minutes and gently shaken on rotary shaker at 70 rpm to ensure that the entire PLB is fully submerged for bacterial adherence onto PLBs. The PLBs were then sieved on a sterile metal sieve and blot-dried on a sterile filter paper to remove excess unattached* Agrobacteria* cells. The infected PLBs were transferred onto modified Vacin and Went media (supplemented with coconut water, 30% tomato extract, 8 g Gelrite, and 200 *μ*M acetosyringone (except in the experiment to optimize acetosyringone concentration); pH 4.8–5.0) and incubated at 28°C for 4 days in the dark for cocultivation. For the control, the PLBs were directly placed on cocultivation medium without being immersed in* Agrobacterium *suspension. At the end of the cocultivation period, the PLBs were detected by histochemical localization of GUS activity.

### 2.5. Histochemical Localization of GUS Activity and Statistical Analysis

The effects of the following parameters known to influence the transformation efficiency were assessed: bacterial density (0.2, 0.4, 0.6, 0.8, 1.0, and 1.0 at OD_600 nm_), wounding level, cocultivation period (1, 2, 3, 4, 5, 6, and 7 days), immersion time (10, 20, and 30 min), and acetosyringone concentration (0, 50, 100, 150, 200, and 250 *μ*M) added to cocultivation media. All the parameters were optimized by screening for transient GUS expression using histochemical localization of GUS activity. All experiments were carried out with 10 samples and repeated six times. The statistical analyses were performed using SPSS 20.0 (SPSS Inc., USA). GUS assay was carried out according to the method described by [29] with slight modification. After being cocultured in the dark for three days, PLBs were incubated in a solution containing 100 mM Na_3_PO_4_ (pH 7.0), 10 mM EDTA, 0.5 mM K_3_Fe (CN)_6_, 0.5 mM K_4_Fe (CN)_6_, 1 mg/mL 5-bromo-4-chloro-3-indolyl-*β*-d-glucuronic acid (X-Gluc), and 0.1% Triton X-100 at 37°C for 48 hours. The stained tissues were then transformed into 75% ethanol for 24 hours to remove chlorophylls. Nontransformed PLBs were used as control.

### 2.6. Optimized *Agrobacterium*-Mediated PLB Transformation

Wounded 3-4 mm PLBs were inoculated with* Agrobacterium tumefaciens *strain LBA4404 containing pW1B1 and pW2KY carrying* wwin1 *and* wwin2* genes, respectively, and* nptII* genes in LB broth supplemented with 50 ppm kanamycin and 100 *μ*M acetosyringone for 30 minutes. The density of* Agrobacterium *suspension was 0.8 at 600 nm and cocultivated for 4 days on modified Vacin and Went media (supplemented with coconut water, 30% tomato extract, 8 g Gelrite, and 200 *μ*M acetosyringone; pH 4.8–5.0). After cocultivation, PLBs were transferred selection media (modified Vacin and Went media supplemented with 15% coconut water, 30% tomato extract, 8 g gelrite, 30 ppm geneticin, and 250 ppm cefotaxime (pH 5.0)) in order to isolate putative transformants.

### 2.7. Molecular Analysis of Putative Transgenic Plants

The DNA extraction kit, Genomic DNA Mini Kit (Plant; Geneaid Biotech Ltd., Taipei County, Taiwan) was used to extract the genomic DNA from the samples. The extraction method was based on the protocol provided by the kit. PR4 (*wwin1* and* wwin2*) and* nptII* transgenes from putative transformants were amplified using MyCycler Thermal Cycler (Bio-Rad Laboratories, Inc., USA). DNA of* A. tumefaciens* strain LBA4404 (harbouring PR4 and* nptII* genes) was amplified via colony PCR to serve as the positive control. All amplification products stained with loading dye were separated on 1.2% (w/v) agarose gel.

### 2.8. Statistical Analysis

Data were analyzed using one-way ANOVA in SPSS 20.0 (SPSS Inc., USA). All analyses were performed at a significance level of 5% with the differences contrasted using Tukey's multiple range test.

## 3. Results

### 3.1. Minimal Inhibitory Concentration of Antibiotics

**Figure 3:**
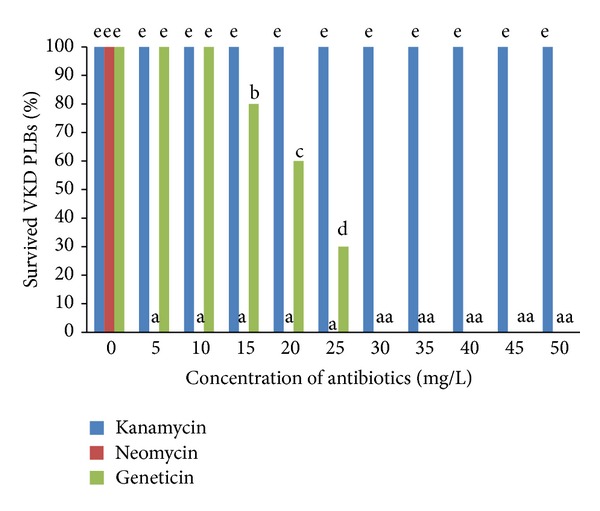
Percentage of survival of the VKD's PLBs after four weeks in selection media containing various concentrations of different antibiotics. Data were analysed using one-way ANOVA and the differences contrasted using Tukey's multiple comparison test. Different letters indicate values which are significantly different (*P* ≤ 0.05).

Nontransformed 12-week-old PLBs were individually isolated and cultured on culture media containing different concentration of antibiotics for four weeks. The percentages of PLBs that survived were plotted against the concentrations of the various selection agents tested (Figure 3) after four weeks.

Kanamycin neither kills the PLBs nor causes browning of tissues even at the highest concentration tested. Kanamycin treatment did not display any toxic effect on PLBs at any tested concentration. PLBs challenged with kanamycin survived the treatment while their survival was undisturbed by kanamycin and comparable to that of control PLBs. PLBs treated with kanamycin at any concentration scored 100% survival at the end of the fourth week (Figure 3). This indicates that PLBs are highly resistant to kanamycin. It shows that kanamycin has the least inhibitory effects on PLBs.

Kanamycin and neomycin were found to be poor selection agents for stable PLBs transformation. Kanamycin allowed the growth of PLBs at higher concentrations while neomycin completely inhibited the growth of PLBs (Figure 3). Neomycin started killing the PLBs at the very low concentration of 5 ppm (Figure 3) compared to geneticin, which only started killing the immature embryos at a concentration of 15 ppm (Figure 3). PLBs treated with neomycin at lower concentrations (5, 10, 15, and 20 ppm) undergone degreening of the tissues and slowly approaching browning stage during the four weeks of observation whereby the development of the PLBs was fully retarded or inhibited. PLBs turned brown indicating tissue death and no regeneration could be observed. This result indicates that neomycin is highly toxic to the survival of PLBs than kanamycin and geneticin.

Geneticin was found to be the best selection agent for PLBs transformation as it inhibits the growth of PLBs in the early stages with lower concentration. Geneticin has effectively killed PLBs at 30 mg/L. Selection using geneticin at a concentration of 15, 20, and 25 ppm significantly reduced the survival of PLBs to 74%, 58%, and 26%, respectively (Figure 3). At a concentration of 30 ppm and above tissues begin to degreen from second week onwards and gradually turned brown and completely died at the end of the forth week. Meanwhile, PLBs treated with geneticin at a concentration of 5 and 10 ppm remained viable.

### 3.2. Optimization of *Agrobacterium*-Mediated PLB Transformation

#### 3.2.1. PLB Size

**Figure 4:**
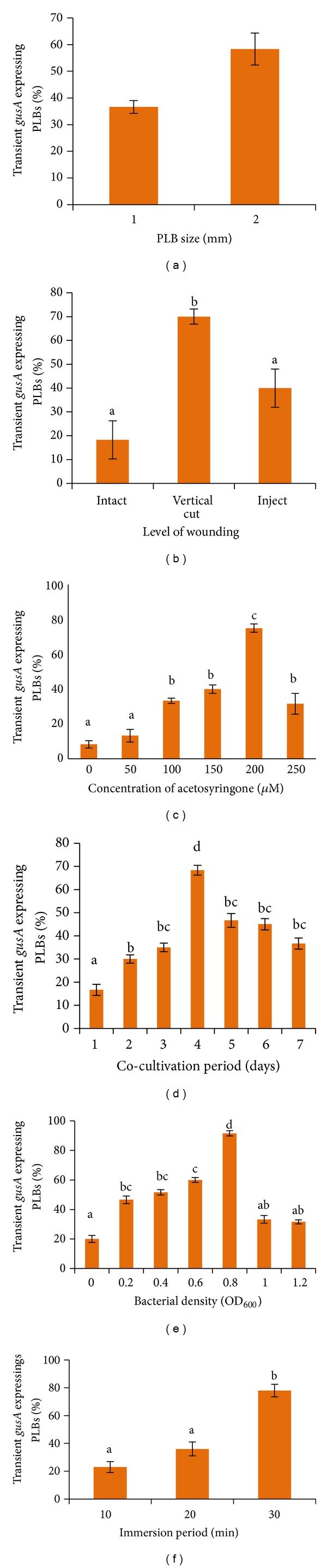
Optimization of the* Agrobacterium*-mediated transformation based on transient* gusA* expression on VKD's PLBs. (a) PLB size; (b) wounding; (c) concentration of acetosyringone; (d) cocultivation period; (e)* Agrobacterium* density; and (f) Immersion period. Results were analysed by one-way ANOVA and means were compared by Tukey's test. Vertical bars represent ± SE of means of 6 replicates. Different letters indicate values which are significantly different (*P* ≤ 0.05).

In this study, 4- to 12-week-old single PLBs, measuring 1-2 mm and 3-4 mm (diameter width) size ranges, were subjected to infection by* A. tumefaciens* suspension culture. The results showed that PLB of 3-4 mm size range gave the highest transient* gusA *expression (58.33%) while the 1-2 mm size range PLB gave the lowest expression (36.6%) (Figure 4(a)). PLBs of 3-4 mm size range were chosen as the target size for subsequent experiments to avoid low survival rate of infected explants. Smaller PLBs size of 1-2 mm turned brown due to the necrosis caused by overinfection of* A. tumefaciens* while PLBs above 3-4 mm of diameter width form clumps, produce secondary PLBs, or begin shooting.

#### 3.2.2. Wounding

Wounding the explants before inoculation was found to enhance transient GUS expression. The highest percentage of GUS expression (70%) was observed on PLBs wounded by scalpel.  Figure 4(b) shows wounding with scalpel significantly (*P* < 0.05) increased the efficiency of PLBs transformation. The study shows that transient GUS activity decreased to 40% when PLBs were injured by needle. Furthermore mild wounding with needle is not recommended for VKD PLBs since there is no significant difference between PLBs injured with needle and intact PLBs.

#### 3.2.3. Acetosyringone

In this study, six concentrations of acetosyringone (0, 50, 100, 150, 200, and 250 *μ*M) were incorporated into cocultivation medium to analyze the effect of acetosyringone in* Agrobacterium*-mediated transformation. The results revealed that* Agrobacterium*-mediated transformation of PLBs occurred both in media supplemented with acetosyringone and acetosyringone-free media. As shown in Figure 3(c), PLBs could be transformed by* Agrobacterium* in the absence of acetosyringone, but the efficiency was low, suggesting that only insignificant amounts of* vir*-specific endogenous phenolic inducers were released. Inclusion of acetosyringone in medium significantly promoted the transient GUS expression of PLBs. The transformation frequency increased from 40 to 75% when the acetosyringone concentration was increased from 150 to 200 *μ*M (Figure 4(c)). Thus, it has proven that the addition of acetosyringone dramatically increased GUS expression. Increasing the concentration of acetosyringone above 200 *μ*M did not appear to further increase transformation frequency and had a negative effect on the transformation of VKD PLBs. GUS expression reduced from 75% to 32% when acetosyringone concentration increased to 250 *μ*M (Figure 4(c)). Concentration above 200 *μ*M was found unsuitable due to a high degree of tissue browning and mortality of PLBs.

#### 3.2.4. Cocultivation

Based on the results obtained, cocultivation period of 4 days produced the highest transient* gusA *expression (68.3%) on VKD PLBs while 1 day cocultivation period scored the lowest transformation frequency which was 16%. However, there was no statistical difference among 2, 3, 5, 6, and 7 days of cocultivation while a steady decrease in transformation frequency was observed after 4 days of cocultivation (Figure 4(d)).

#### 3.2.5. Bacterial Density

Transformation efficiency influenced by* Agrobacterium* density in suspension form was investigated by measuring optical density at the wavelength of 600 nm (OD_600 nm_). Differences in the transient GUS expression were observed for each level of bacterial density. The suspension culture of the* Agrobacterium *with OD_600 nm  _0.8 produced the highest number of GUS positive explants which scored 91.6%, followed by 0.6 and 0.4 scoring 60% and 51.6%, respectively (Figure 4(e)). Results showed that there is a significant difference between OD_600 nm  _ 0.6 and 0.8. It was concluded that the optimal bacterial density for VKD's PLBs is 0.8 at OD_600 nm_. Nevertheless, there is no significant difference among results obtained for* Agrobacterium *suspension at OD_600 nm  _ 0.2, 0.4, 1.0, and 1.2. OD_600 nm  _ 1.0 and 1.2 reduced the transient GUS expression to 33.3 and 31.6%. A denser* Agrobacterium* suspension (OD_600 nm  _ of 1.0 and 1.2) will allow maximum bacterial attachment above the optimal level. Furthermore, the* Agrobacterium* suspension used in this study was under early-log phase (bacteria obtained from cultures grown for 16 hours) and they were actively dividing cells. Thus,* Agrobacterium* suspension with OD_600 nm  _ 1.0 and 1.2 is not suitable for transformation studies because it may cause necrosis on PLB tissues.

#### 3.2.6. Immersion Time

The frequency of* gusA* expressing VKD PLBs was 23.3% and 33.6% when the infection period was 10 and 20 minutes, respectively, which is lesser compared to 30 minutes. Results indicated that 30 minutes was optimum for transforming VKD PLBs (Figure 4(f)). Since there is a significant difference (*P* < 0.05) between treatments, 30 minutes was chosen as the immersion time in order to get highest transformation efficiency (Figure 4(f)).

### 3.3. Detection of Transgenes in Transgenic Lines Using PCR Analysis

**Figure 5:**
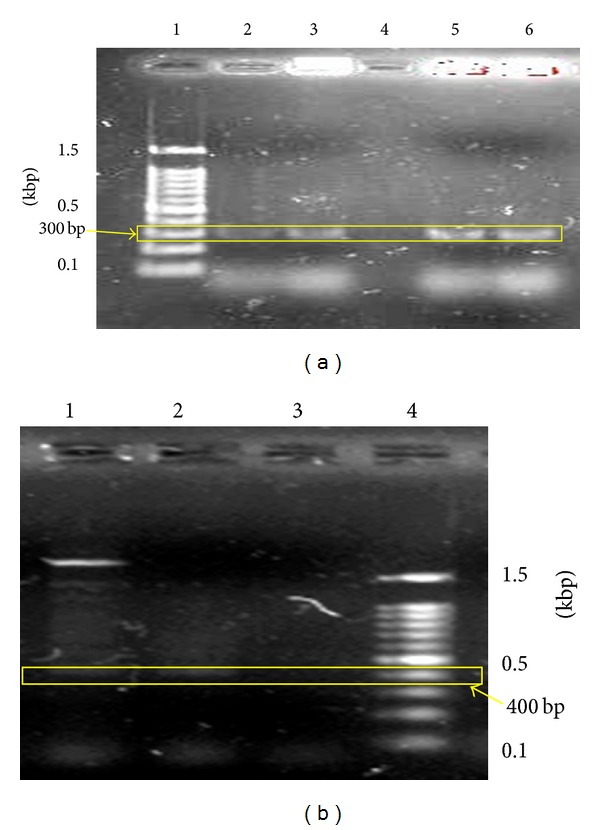
Molecular analysis of transgenesintegration in putative transgenic plantlets and control PLBs. (a) Single band of 300 bp which was produced on lanes 2 and 3 confirmed the transfer of* wwin1* and* wwin2* genes (*pr4* genes) into the PLBs (lane 1, marker; lane 2, putative transformant PLB (pW1B1); lane 3, putative transformant PLB (pW2KY); lane 4, untransformed PLB (control); Lane 5,* A. tumefaciens *(pW1B1); lane 6,* A. tumefaciens *(pW2KY)); (b) single band of 400 bp was produced on lanes 5 and 6 confirmed the transfer of* nptII *genes into the PLBs (lane 1, putative transformant PLB (pW1B1); lane 2, putative transformant PLB (pW2KY); lane 3, untransformed PLB (control); lane 4, marker).

Selection process on selection media containing cefotaxime and geneticin produced 82% and 68% recovery rate for PLBs cocultivated with* A. tumefaciens *strain LBA4404 harbouring plasmids pW2KY and pWIBI, respectively.  Figure 5(a) shows the band separation of* wwin* gene from respectable samples. Lane 1 contained the 100-bp DNA ladder (Fermentas, USA) for reference purpose. Lane 2 and 3 contained the PCR products from PLBs transformed with* A. tumefaciens* strain LBA4404 carrying* wwin1* and* wwin2* genes, respectively. A single band of 300 bp was observed on lanes 2 and 3 containing PCR products from the putative transformants. Lane 4 produced no band since it contained the PCR products of negative control which is the nontransformed PLB. Lanes 5 and 6 contained the PCR products of* A. tumefaciens* strain LBA4404 carrying* wwin1* and* wwin2* genes, respectively. A single band of 300 bp was also observed for the PCR products of* A. tumefaciens*. This shows that VKD PLBs have successfully transformed using* A. tumefaciens* strain LBA4404 with* wwin1* and* wwin2 *genes.

Figure 5(b) shows the PCR analysis of* nptII *gene extracted from the putative transformants and control PLB. Lane 4 contained the 100-bp DNA ladder (Fermentas, USA) for reference purpose. No band was observed on lane 3 which contained the PCR products of untransformed control PLB. Single band of 400 bp was scored on lanes 1 and 2 containing PCR products of PLBs transformed by* A. tumefaciens* strain LBA4404 carrying* nptII* gene. The presence of* nptII *gene in putative transformants confirmed the successful transformation event and supports the observation that transformed PLBs survived on the selection media containing geneticin.

## 4. Discussion

### 4.1. Determination of the Minimal Inhibitory Concentration of the Selection Agents

Antibiotics differ in stringency depending upon their mode of action that ultimately decides its value for the selection of transformants [30]. Based on the result, geneticin was selected as the most suitable selection agent. PLBs challenged with geneticin begun to completely die from 30 ppm onwards. Thus, transformants that express* nptII* gene will allow the recovery of transgenic PLBs with no signs of necrosis in the presence of 30 ppm geneticin. Shin and team observed sharp decline in fresh weight of sweet potato callus at 5 and 10 ppm geneticin and recorded markedly lower cell viability at greater concentrations of geneticin [31]. On the other hand, ineffectiveness of geneticin has been reported previously for maize [32] and oil palm [33]. The variation in the sensitivity of monocots towards geneticin could be due to the difference in endogenous resistance [21].

Kanamycin and neomycin were found to be poor selection agents for stable PLBs transformation. Neomycin completely inhibited the growth of untransformed PLBs even at the lowest concentration (Figure 3). This indicates that PLBs showed extreme sensitivity towards neomycin that completely arrested the growth of untransformed tissues. Contrarily, single buds of banana cultivar Rastali (AAB) were insensitive to neomycin and required as high as 300 ppm of neomycin to completely inhibit the regeneration of explants after 24 days [34]. Moreover, neomycin had been proved for stimulatory effect on the regeneration of apple tissue [35].

Contrarily, VKD's PLBs do not express any signs of toxicity and remain viable at the highest concentration of kanamycin (50 ppm). Many crops are resistant to kanamycin, making it inefficient for the selection of putative transformed plants by allowing escapes [31]. Endogenous resistance due to the inability of kanamycin to be transported through the cell wall suggests usage of higher concentration of kanamycin for selection process. For instance, kanamycin concentration above 3000 ppm is required to totally inhibit the growth of oil palm immature embryos [21]. However, elevated concentration of antibiotics is not advisable because it may kill off the putative transformants that received small number of transgenes, is economically unfeasible and biologically ineffective.

### 4.2. Optimization of Parameters Influencing the Efficiency of *Agrobacterium*-Mediated Transformation

Several factors known to enhance the* Agrobacterium*-mediated transformation were optimized based on GUS expression. A number of factors such as PLB size, level of wounding, concentration of acetosyringone, cocultivation period,* Agrobacterium *density, and immersion period were studied to improve the* Agrobacterium*-mediated transformation of VKD PLBs.

In this study, 4-week-old single PLB, measuring 1-2 mm, and 12-week-old single PLB, measuring 3-4 mm (diameter width) size ranges, were subjected to infection by* A. tumefaciens* suspension culture. Based on the result, individual PLBs of 3-4 mm size produced the highest transient GUSexpression (Figure 4(a)). Thus, PLBs of 3-4 mm size were chosen for the subsequent optimization and transformation studies. Recovery of transformed tissues is not possible for the PLBs of 1-2 mm. Smaller PLBs have the tendency to die of necrosis caused by infection of* A. tumefaciens*. Furthermore, the aim of producing transgenic orchid plantlet will be hampered. On the other hand, PLBs above 3-4 mm of diameter width form clumps, produce secondary PLBs, or begin shooting. Hence, they cannot serve as a suitable target explants for* Agrobacterium*-mediated transformation.

Wounding is an integral step in the* Agrobacterium*-mediated transformation because acetosyringone released from injured part plays chemotactic role and induces the* vir* genes to initiate T-DNA transfer [36, 37].  Figure 4(b) shows that wounding with scalpel produced the highest percentage of transient GUS expression. Severe wounding using scalpel injures the epidermal and subepidermal layers of PLB. Hence, large numbers of bacteria will colonize the epidermal region and penetrate deeper within the wounded tissue. This will enhance the transfer of a foreign gene from* Agrobacterium* to PLBs. Mild wounding with needle is not recommended for PLBs. Perhaps, similar to intact PLBs, mild wounding does not produce copious amount of phenolics to chemotactically attract* Agrobacterium *cells.

Orchids are not the natural hosts of and recalcitrant to* Agrobacterium*. Transformation efficiency of orchids can be improved by the addition of acetosyringone at various concentrations during infection as well as subsequent cocultivation stages [8]. Successful GUS expression on exogenous acetosyringone-free treated PLB (Figure 4(c)) shows that PLB has the capability to produce phenolics endogenously. However, the level is sufficient to chemotactically attract* Agrobacterium* cells but too low to elicit successful* vir* gene activation. Similarly, PLBs treated with lower concentration of acetosyringone produced lower GUS expression (Figure 4(c)). PLBs treated with 200 *μ*M acetosyringone scored the highest transient GUS expression (Figure 4(c)). Thus, inclusion of 200 *μ*M acetosyringone to the cocultivation medium reduces the recalcitrant effect of VKD orchid PLB. Hence, orchid PLBs were made to mimic the natural host of* Agrobacterium* to allow transformation of PLB.

Although addition of acetosyringone significantly enhanced the GUS expression, increasing the concentration of acetosyringone above supraoptimal concentration (200 *μ*M) proportionally increases the browning of PLB tissues (Figure 4(c)). Browning is a sign of necrosis and indicates excessive colonization of* Agrobacterium* on PLBs. Similarly, transformation of cauliflower [38] and* Dioscorea zingiberensis* Wright [39] was adversely affected because of higher concentration acetosyringone. Thus, addition of acetosyringone above 200 *μ*M will produce detrimental effect on the PLB and prevent a successful transformation event.

Duration of immersion and cocultivation have significant effect on transformation efficiency. Although 2-3 days of cocultivation is standard for most transformation protocols [11], VKD PLBs proved that it requires a longer cocultivation period. Shorter cocultivation period ranging from 1 to 3 days was not sufficient for* Agrobacterium*-mediated transformation of VKD PLBs. Shorter cocultivation period restricts* Agrobacterium* from collapsing the physical barrier on plant tissues to access for transgene transfer on the intact PLBs. PLBs cocultivated for 4 days produced the highest transient GUS expression. This shows that 4 days of cocultivation is sufficient for the successful induction of virulence, chemotaxis, attachment, and transgene transfer. Negative influence of longer cocultivation period was observed in terms of reduced GUS expression and occurrence of dead cells. Similarly,* Phalaenopsis* calli underwent necrosis and died when the cocultivation period was too long [14]. Overgrowth of bacteria leading to explant necrosis and death is a major drawback in prolonged cocultivation [40, 41]. Therefore, cocultivation period should be optimized to achieve highest transformation efficiency, but least necrosis of transformed tissues.

In* Agrobacterium*-mediated transformation, target tissues are infected with fresh overnight suspension culture of bacteria. PLBs treated with* Agrobacterium *suspension with OD_600 nm  _0.4, 0.6, and 0.8 produced transient gene expression above 50% in a steadily increasing order. However, statistically significant OD_600 nm  _ 0.8 was selected to further the transformation studies (Figure 4(e)). Although denser* Agrobacterium* suspension (OD_600 nm  _ 1.0 and 1.2) allows maximum bacterial attachment onto PLBs, it may cause contamination by* Agrobacterium* itself. Eventually PLBs will undergo irreversible physiological disturbances that lead to browning of tissues and unsuccessful recovery of transformed cells [42]. Increased bacterial infectivity may lead to hypersensitive response of explants to bacteria and cause reduction of regeneration frequency [43]. On the other hand, transformation efficiency was low in OD_600 nm  _ 0.2 due to the fact that there is a lack of sufficient* Agrobacterium* cells to infect and transfer T-DNA into PLBs [44].

Examination on immersion time indicated that 30 minutes was optimum for transforming VKD's PLBs (Figure 4(f)) although 10 and 20 minutes produced appreciable level transient GUS expression. Long immersion period allows more bacteria to get adhered onto the surface of PLB for a better chance of inserting transgene into the plant genome. Lengthy immersion period may also allow the formation of* Agrobacterium *biofilm on PLB surface which may be responsible for the 78% of transient GUS expression (Figure 4(f)). Previously it was reported that 30-minute immersion period resulted in higher transformation efficiency compared to longer immersion periods of 45 minutes and 60 minutes [11]. A combination of shorter immersion period and physical force such as rotation on the shaker may have prevented the irreversible attachment of* Agrobacterium* onto PLBs, hence reducing the GUS expression on PLB treated with shorter immersion period.

### 4.3. Molecular Analysis of the Putative Transformants

DNA extracted from the putative transformants produced a single band of 300 bp (Figure 5(a)) and single band of 400 bp (Figure 5(b)). Presence of the same bands at the 300 bp and 400 bp by the DNA extracted from* A. tumefaciens* proves that VKD PLBs were successfully transformed by* A. tumefaciens* strain LBA4404 to express PR4 and* nptII* genes.

## 5. Conclusion

A simplified procedure for* Agrobacterium*-mediated transformation has been designed for VKD PLBs. The putative transformants isolated by selection via inclusion of 30 ppm geneticin in selection media are capable of producing antifungal protein (PR4) to either tolerate or resist the fungal disease at enhanced level. In summary, this present study revealed that the parameters including PLB size, cocultivation period, immersion period, concentration of acetosyringone, wounding level of PLB, and* Agrobacterium* density are critical to achieve high transformation rates. The improved VKD transformation system described here is reliable, suited for small-scale as well as large-scale transformation experiments generating a large number of transgenic lines.

## Abbreviations

VKD: * Vanda* Kasem's Delight
PLB: Protocorm-like body.
